# Forest conversion into pasture selects dung beetle traits at different biological scales depending on species pool composition

**DOI:** 10.1002/ece3.9950

**Published:** 2023-04-24

**Authors:** Marcelo Bruno Pessôa, Tatiana Souza do Amaral, Paulo De Marco Júnior, Joaquín Hortal

**Affiliations:** ^1^ Departamento de Ecologia, Instituto de Ciências Biológicas Universidade Federal de Goiás Avenida Esperança s/n, Campus Samambaia, ICB 5 CEP 74690‐900 Goiânia Brazil; ^2^ Laboratório de Entomologia Universidade Federal de Mato Grosso do Sul Câmpus de Chapadão do Sul – Rod MS 306, Km 105 CEP 79560‐000 Chapadão do Sul Brazil; ^3^ Department of Biogeography and Global Change Museo Nacional de Ciencias Naturales (MNCN‐CSIC) C/José Gutiérrez Abascal 2 28006 Madrid Spain

**Keywords:** Atlantic Forest, Cerrado, community structure, functional diversity, habitat structure, land transformation, Scarabaeinae dung beetles, trait selection

## Abstract

1. The conversion of forests into open areas has large effects on the diversity and structure of native communities. The intensity of these effects may vary between regions, depending on the existence of native species adapted to open habitats in the regional pool or the time since habitat change.
2. We assess the differences in species richness and functional diversity of dung beetle communities (Coleoptera: Scarabaeinae) between native forests and novel pasturelands of the Atlantic Forest and the Cerrado, two biomes with contrasting histories of human occupation in Brazil. We conducted standardized surveys in seven forest fragments and adjacent pastures in each region and measured 14 traits in individuals collected in each type of habitat at each particular site. We calculated functional richness, functional evenness, functional divergence, and community‐weighted mean of traits for each area, and analyzed individual variation through nested variance decomposition and Trait Statistics.
3. Communities were richer and more numerous at the Cerrado. We did not find any consistent relationship between functional diversity and forest conversion beyond the changes in species diversity. Although landscape changes were more recent at the Cerrado, the colonization of the new habitat by native species already adapted to open habitats lessens the functional loss in this biome. This indicates that habitat change's effects on trait diversity depend on the regional species pool rather than on time since land conversion.
4. Forest conversion effects were primarily due to internal filtering. The effects of external filtering only appear at the intraspecific variance level, with contrasting differences between the Cerrado, where traits related to relocation behavior and size are selected, and the Atlantic Forest, where selection operates for traits related to relocation behavior and flight. These results evidence the importance of considering individual variance to address the responses of dung beetle communities to forest conversion.

## Graphical abstract

Briefly, we assess the impacts of forest conversion on the functional diversity of dung beetles in two different regions of Brazil, considering in particular intraspecific variation. To do this we conducted standardized surveys in seven forest fragments and adjacent pastures for every region and measured fourteen traits in individuals collected in each type of habitat (pasture and forest) at each particular site. We calculated functional richness, functional evenness, functional divergence, and community‐weighted mean of traits for each area, and analyzed individual variation through nested variance decomposition and Trait Statistics. We found that the impact of forest conversion is context‐dependent, since communities were richer and more numerous at the Cerrado, although we did not find any relationship between functional diversity and forest conversion, except when considering intraspecific variance. Further, forest conversion effects were primarily due to internal filtering. External filtering effects only appear when intraspecific variance is considered.

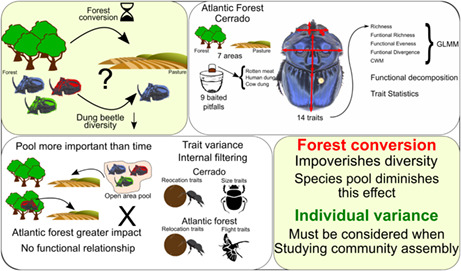

## Resumo

1. A conversão de florestas em áreas abertas tem grandes efeitos sobre a diversidade e estrutura das comunidades nativas. A intensidade desses efeitos pode variar entre regiões, dependendo da existência de espécies nativas adaptadas a habitats abertos no pool regional ou do tempo de mudança de habitat.
2. Avaliamos as diferenças na riqueza de espécies e diversidade funcional de comunidades de rola‐bosta (Coleoptera: Scarabaeinae) entre florestas nativas e pastagens da Mata Atlântica e do Cerrado, dois biomas com histórias contrastantes de ocupação humana no Brasil. As comunidades foram amostradas em sete fragmentos florestais e pastagens adjacentes de cada região. Medimos quatorze caracteres de indivíduos coletados em cada ponto amostral de cada habitat e região. Posteriormente, calculamos a riqueza funcional, a equidade funcional, a divergência funcional e a média dos caracteres ponderada pela comunidade (CWM) para cada ponto amostral e analisamos a variação individual por meio de decomposição de variância aninhada e estatística T.
3. As comunidades do Cerrado foram mais ricas e numerosas. Não encontramos nenhuma relação consistente entre diversidade funcional e conversão florestal além das mudanças na diversidade de espécies. Surpreendentemente, embora as mudanças na paisagem tenham sido mais recentes no Cerrado, a colonização do novo habitat por espécies nativas já adaptadas a habitats abertos diminuiu a perda funcional nos novos habitats deste bioma em comparação com a Mata Atlântica, onde as pastagens foram colonizadas apenas por espécies florestais generalistas e invasores exóticos. Isso indica que os efeitos da mudança de habitat na diversidade de caracteres dependem do conjunto regional de espécies e não do tempo desde a conversão da paisagem.
4. Além disso, os efeitos da conversão florestal foram principalmente devidos à efeitos de filtros internos. Os efeitos de filtros externos apareceram apenas no nível de variância intraespecífica, com diferenças contrastantes entre o Cerrado, onde são selecionados caracteres relacionados ao comportamento de realocação e tamanho (Comprimento da Metatíbia, Comprimento, Volume e Largura do Pronoto) e a Mata Atlântica, onde a seleção opera em caracteres relacionadas ao comportamento de realocação e voo (Metatibia Length e Wing Load). Esses resultados evidenciam a importância de se considerar a variância individual para abordar as respostas das comunidades de rola‐bostas à conversão florestal.

## 1 INTRODUCTION

Forest conversion is a major threat to biodiversity in tropical landscapes (Newbold et al., 2015). The conversion to open areas has large effects on native communities, through changes in habitat structure, the exclusion of native species, and the facilitation of invasions. Such replacement of native species by aliens may affect ecosystem functioning and decrease the effectiveness of the community in utilizing resources and resisting other disturbances (Harrison et al., 2014). Trait‐based functional diversity can provide means to assess these effects on the biodiversity–ecosystem functioning relationship (Flynn et al., 2011).

Functional diversity can be measured from the range of variation in the functional traits of the species present in the community, assuming that ecological functioning can be indirectly assessed through the diversity of traits with functional meaning (Díaz & Cabido, 2001). Within this framework, a functional trait is any measurable characteristic of the individual (morphological, biochemical, phenological, physiological, and behavioral) that affects either its fitness, the fitness of other individuals of the same or different species, or other abiotic ecosystem processes (Violle et al., 2007). Traits are used under the assumption that these characteristics provide information on the ability of individuals to perform particular functions and/or respond to biological interactions, thus providing a good proxy for ecological functionality. Therefore, by measuring different aspects of trait variation, different indices of functional diversity are thought to account for different aspects of functioning (Mason et al., 2005): Functional richness measures the functional (i.e., trait) space occupied by the species in the community; functional evenness does so for the regularity in the use of this space; and functional divergence accounts for how the differences in the distribution of the species in the trait space, which may contribute to better use of resources.

The use of traits in functional ecology is less common—though increasing—for animals than for plants and has been mostly focused on the study of assembly processes (Moretti et al., 2017). One common approach to conceptualize the assembly process is community filtering, where a series of filters determine which species are able to colonize the focal habitat fragment or locality and survive there. These filters are commonly divided into two categories: environmental (i.e., abiotic conditions) and biological (including competition, facilitation or density‐dependent processes). Although this approach widely used, using it poses some challenges, as some biological filters may affect environmental filters, and vice versa. This led to the proposal of external and internal filtering processes (Violle et al., 2012). External filtering would select species from the pool on a scale larger than the community, through environmental or biological factors such as large‐scale climatic gradients, or predator pressure along the landscape. Whereas internal filtering would encompass processes occurring locally within the studied community, like density‐dependent processes or microclimatic heterogeneity. These two types of filters are relative to the spatial scale of the community of interest, so this approach helps overcoming the complex interpretration of traditional filters, whose effects are often impossible to separate one from another (Violle et al., 2012). Nonetheless, this approach allows evaluating the scale at which the largest effects on the community are occurring through intraspecific trait variation. Using only mean trait values per species does not allow assessing, for example, the effects on individuals who have trait values around the optimal mean, and can be benefited from density‐dependent processes such as competition, increasing their fitness in the community. In this case, internal filters can increase variability by reducing the competitive pressure on these individuals, affecting the distribution of trait values around the mean optimal value selected by the external filter (Turcotte & Levine, 2016).

It is important to highlight the reduced number of experiments assessing trait functionality in animal functional ecology (see Noriega et al., 2018 for insects). Dung beetles (Coleoptera: Scarabaeinae) are to some extent an exception to this, being one of the few groups where several of these experiments had been carried out (deCastro‐Arrazola et al., 2020; Emlen et al., 2005; Macagno et al., 2016; Nervo et al., 2014; Slade et al., 2007; but see deCastro‐Arrazola et al., 2023). Indeed, dung beetles can inform about the processes involved in the responses to forest conversion. They are a good model for these studies because they present rapid responses to ecological changes and are easy to collect (Gardner et al., 2008; Nichols et al., 2007). Dung beetles are well‐known for their feeding on mammal feces and the behavior of making and rolling dung balls shown by some of them (Halffter & Matthews, 1966). Their most iconic function is dung removal, but they also provide other functions such as parasite and fly control, soil bioturbation, contribute to diminishing CO_2_ emission in pastures, incorporate NO_3_ in the soil, and act as secondary dispersal of seeds and enhance plant growth (deCastro‐Arrazola et al., 2023; Nichols et al., 2008; Slade et al., 2016). Their distinct feeding behaviors provide a classification in guilds that provides a rapid approach to their functional diversity (Doube, 1990; see also Pessôa et al., 2017). They can be classified as Rollers that make a dung ball and roll away; Tunnelers that burrow the dung; and Dwellers that live directly in the dung (Bornemissza, 1969; see also Tonelli, 2021). This knowledge of their natural history may help to better interpret the patterns observed in nature.

In the case of Neotropical dung beetles, the conversion of forest into pasture affects community structure by diminishing their richness and increasing the dominance of a few species (Nichols et al., 2007; Sánchez‐de‐Jesús et al., 2016). In functional terms, forest conversion affects dung beetle food relocation behavior, body size and daily activity (i.e., diurnal, nocturnal, or crepuscular) (Nichols et al., 2013), as well as their effects on ecosystem service provision (Noriega, March‐Salas, et al., 2021). Although the effects of land‐use change on the spatial and temporal dynamics of Neotropical dung beetle communities are relatively well‐known (Dale et al., 1994; Gardner et al., 2008; Klein, 1989; Korasaki et al., 2013; Lopes et al., 2011; Noriega, Santos, et al., 2021), there is a need for a better understanding of their responses considering their evolution in more forested areas, in comparison with Afrotropical and Palearctic regions. Functional diversity indices can inform about these responses, and dung beetle functional richness and divergence decrease as the impact of forest conversion increases (Barragán et al., 2011).

The Atlantic Forest and Cerrado biomes are both biodiversity hotspots, and their biotas are the result of distinct evolutionary histories and ecological processes that, arguably, have resulted in different regional pools of species. In general, dung beetle diversity in the tropics is greater in the forests than in open areas (Hanski & Cambefort, 1991; but see Silva et al., 2014). However, their ecological particularities create a conspicuous difference in the diversity of both biomes (Durães et al., 2005), since the natural landscapes of the Cerrado (aka. the Brazilian Savannah) host more natural open areas than those of the Atlantic Forest. Furthermore, the history of forest conversion in Brazil is spatially uneven (Leite et al., 2012). The Atlantic Forest was one of the first areas to be converted, mostly because it is situated in the coastal region, providing easy access to European settlers (Dean, 1997). Whereas the Cerrado was exploited more intensively in the expansion and internalization of the Brazilian population promoted by President Getulio Vargas in the 1950s (Oliveira & Marquis, 2002). Given the contrasting ecology and history of these two biomes, we expect that the differences in the functional adaptations evolved by dung beetles at each one of them would also affect their ability to colonize the novel open habitats.

We evaluate the effects of forest conversion into pasture on the functional diversity of dung beetle communities in the Atlantic Forest and the Cerrado. More specifically, we use data on community composition and trait measurements gathered from standardized surveys of forest fragments and pastures from seven landscapes within each biome, to evaluate whether forest conversion selects particular functional traits of dung beetles in each region through functional diversity indices and trait variations both between and within species. Therefore, we aim to answer the following questions: (1) How does forest conversion in the Atlantic Forest and the Cerrado affect the richness and functional structure of dung beetle communities? We expect that the effect in richness will be stronger than in the functional structure since dung beetles have a high functional redundancy; and that the time of conversion may decrease this effect (the Atlantic forest conversion event was about 100 years ago, whereas in the Cerrado, conversion happened roughly 40 years ago). (2) Is there a shift in the values of functional traits in the novel habitats created by the forest conversion? Here, we expect that traits related to dispersion or food reallocation will show larger values in pastures since the resource is more exposed, while in the forest traits related to maneuverability will be more important since the forest presents more obstacles during flight. (3) Which scale represents the variance found in the traits studied? We expect that individual variations (i.e., intraspecific variance) and internal filters will be most important in the habitats with greater competition intensity.

## 2 MATERIALS AND METHODS

### 2.1 Study areas

**FIGURE 1:**
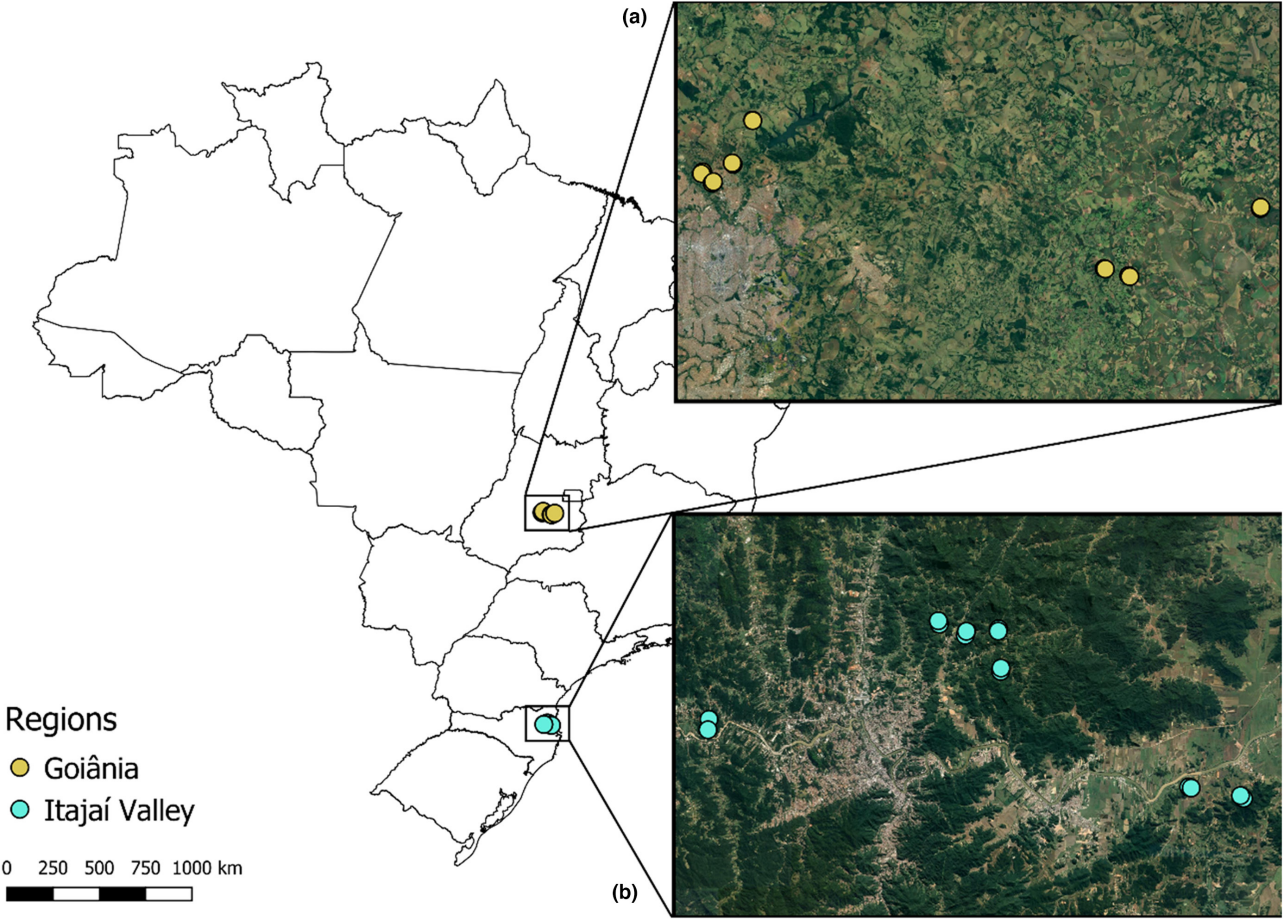
Location of the regions and areas of the dung beetle surveys. (a) Goiânia region—Cerrado. (b) Itajaí Valley region—Atlantic Forest.

This study was carried out in two different regions of Brazil: the Itajaí Valley (Santa Catarina) and the surroundings of Goiânia (Goiás) (Figure 1). The Itajaí Valley is part of the Atlantic Forest biome, an evergreen tropical rainforest that has a constantly humid condition. This biome comprises different vegetation types, such as seasonally semideciduous and deciduous forests, mixed ombrophilpus Araucaria forests, and ombrophilous dense forests (IBGE, 2012). In this study, we selected all fragments in ombrophilous dense forests, a formation characterized by large trees with dense crowns, which can reach 35 m in height, giving rise to a continuous canopy structure, and by a dense shrub understory, formed mainly by shrubs, herbs, and seedlings. There is also a wide variety of epiphytes, consisting of bromeliads, orchids, ferns, mosses, and lichens, resting on the branches of trees and shrubs (Vibrans & Sevegnani, 2013).

Goiânia region is located in the Brazilian Cerrado. This biome is subject to a regular and long drought period, which can last around 6 months. It is characterized by highly heterogeneous vegetation, composed of a continuum of areas of savanna, ranging from open grasslands, with no trees or shrubs (“campo limpo”), to forests (locally known as “cerradão”) (IBGE, 2012). We selected all forest sites in fragments of “cerradão”. This formation is characterized by the predominance of tree species, which can reach from 8 to 15 m or taller, giving rise to a continuous canopy. Many species are deciduous, so the crown cover can vary from 50% to 90% throughout the year. The understory is formed by small shrubs, herbs, and a few types of grass (Sano, 2008). Trees’ crowns cast a considerable shadow, which makes the shrub and herbaceous layer smaller when compared to the other types of formations in the Cerrado.

### 2.2 Dung beetle surveys

**FIGURE A1:**
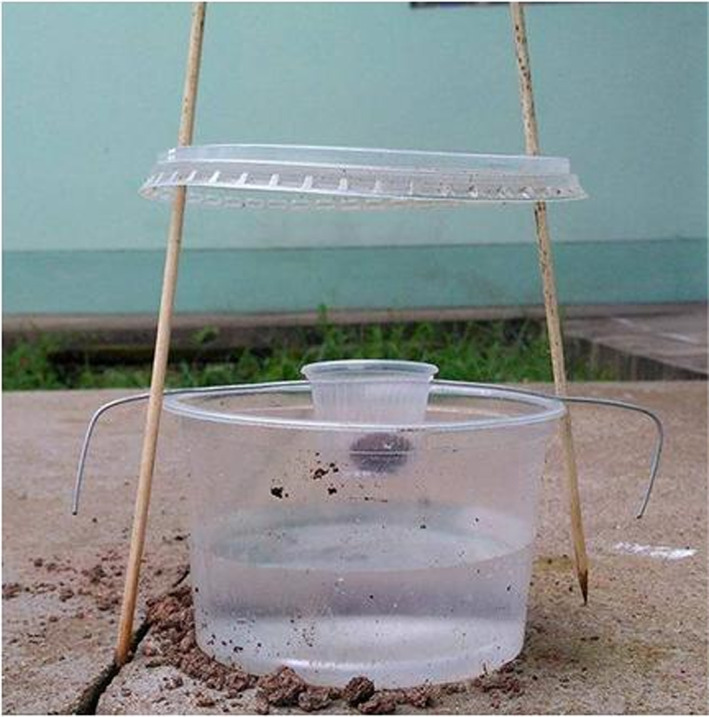
Baited pitfall trap used to collect the dung beetles.

**FIGURE A2:**
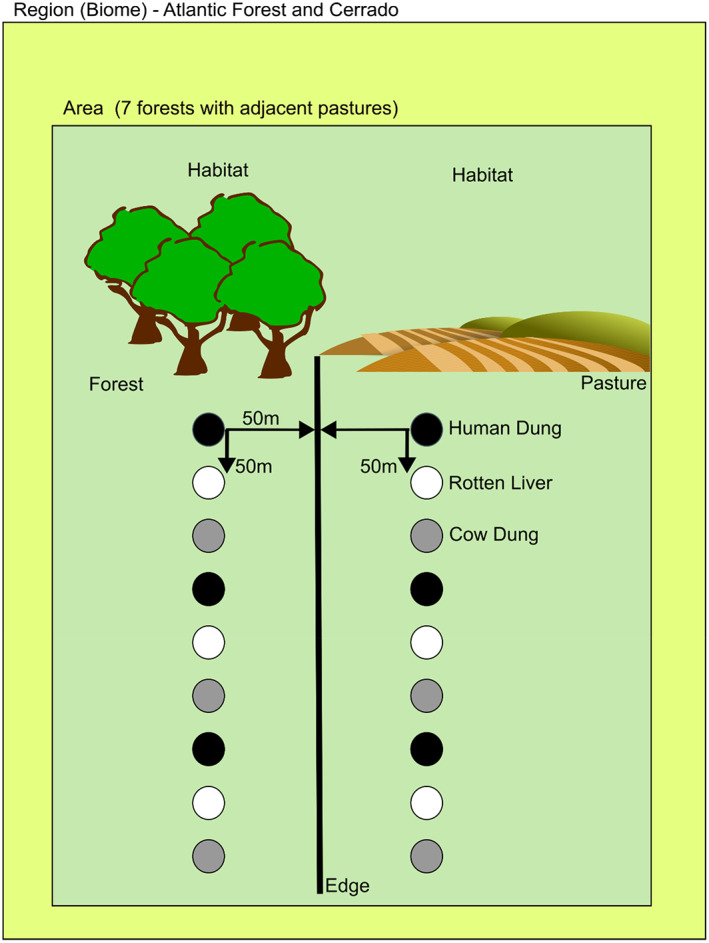
Design of the surveys of the dung beetle communities in two regions (placed at the Atlantic Forest and Cerrado biomes). In each region, seven areas were selected, and in each area, two habitats were sampled: one forest patch and one pasture adjacent to the forest. In each habitat (forest and pasture), nine pitfall traps with three different types of baits (human feces, rotten liver, and cow dung) were placed along a linear transect at 50‐m intervals. In both transects, traps were placed at least 50 m from the habitat edge, to account for edge effects.

In each region, Cerrado and Atlantic Forest, we selected seven areas separated at least 1 km from each other. In each one of those areas, we conducted standardized surveys in two adjacent sites: one of forest and other of pasture. Dung beetle captures were made with baited pitfall traps consisting of 1‐L pots with a solution of water, salt, and detergent. The baits were suspended above the trap with wire in a 50‐mL plastic cup (Figure A1). Three different types of baits were used: human feces, rotten liver, and cow dung. We placed three replicates of each type of bait, so in total, nine pitfall traps were placed in each sampling site, spaced 50 m apart along a transect, and separated at least 50 m from the edge of the fragment (Figure A2). The traps remained for 48 h in both habitats (forest and pasture). We considered each pair of habitats as a sample unit. The surveys were conducted in the rainy seasons of 2016 and 2017. All collected beetles were identified by Fernando Z. Vaz‐de‐Mello (Universidade Federal de Mato Grosso) and deposited in the entomological collection of the Universidade Federal de Goiás. The dung beetle species pool in each region was obtained from the results of our surveys. Fragment size, shape, and conservation status may have some effects on dung beetle communities. We dealt with these undesired effects by surveying pasture and forest fragments in a pairwise manner. Also, we accounted for the particularities of each area by using it as a random factor in the models.

### 2.3 Measuring dung beetle functional traits

We compiled information on a set of functional traits for each species and site based on measurements of the dung beetle individuals collected in the surveys. In total, we selected 15 traits (Table 1) related to: dispersion (wing load, wing area/length ratio, and eye dorsal area; Byrne & Dacke, 2011; Dacke et al., 2013; Hongo, 2010); excavation (prosternum height, protibiae area, pronotum width, head length, and head width; Halffter & Matthews, 1966; Vilhelmsen et al., 2010); resource use (body size, measured as pronotum length + elytra length, and volume measured as length × pronotum width × prosternum height; Andresen, 2003; Emlen et al., 2005; Radtke & Williamson, 2005); food relocation (horizontal displacement and metatibia length; Halffter & Matthews, 1966); breeding behavior (nesting habit and nest shape—pear/ball; Halffter & Matthews, 1966), diel activity (Hernández, 2002); and specialization (i.e., food specificity; Falqueto et al., 2005).

**TABLE 1:** Dung beetle traits selected to measure an their ecological meaning.

	Trait	Abreviation	Biological interpretation	Ecological function
1	Volume	Vol.	Individuals with greater volume and length will need more resource to fully develop	Amount of resource needed to development
2	Length	Len.		
3	Prosternun height	Ps.H	Individuals with higher prosternum will have more muscle mass for front leg use, increasing excavation strength and improving the individual use of different compacted soils	Soil and resource excavation
4	Pronotum width	Pr.W	Individuals with wider pronotum excavate larger tunnels	
5	Protibia area	Pt.A	These traits are direct related with the digging action. So greater values in these traits indicates a greater “shovel” area that increases the individual digging ability	
6	Head length	He.L		
7	Head width	He.W		
8	Eye dorsal area	Ey.A	Greater eye dorsal area means greater visual reception to maneuverability during flight and localization ability	Dispersal
9	Wing load	W.Lo	Greater wing load indicates greater flight ability	
10	Metatibila length	Me.L	Individuals with larger metatibias have increased rolling ability	Food relocation
11	Horizontal displacement	Ho.D	Species that present rolling ability isolate part of the resource and disperses seeds in larger distances	
12	Nesting	Nes	Species that present parental care behavior increase larval success	Parental care
13	Pear/ball nest	Pe.B		
14	Specificity (Levins standardized index)	Le.S	Greater value in this index means that the species have a wider niche	Resource generalism
15	Dial activity	Di.A	This trait represent foraging time of the species	Phenology

**FIGURE A3:**
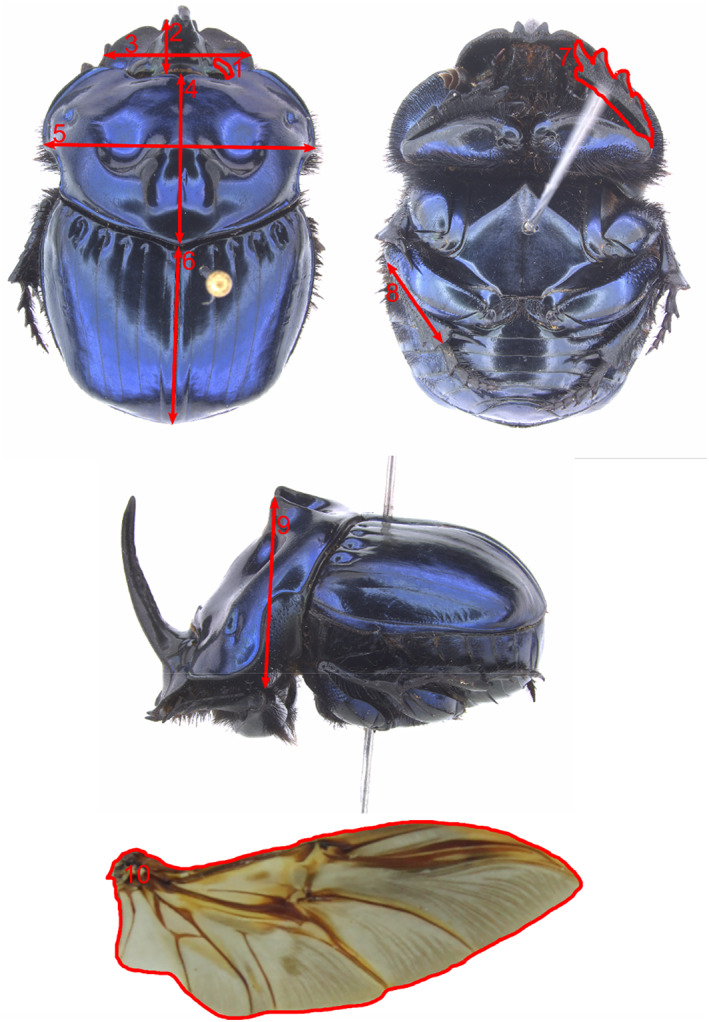
Dung beetle functional traits measured in five individuals per habitat per area. The traits were measured by ImageJ. 1. Dorsal eye area, 2. Head length, 3. Head width, 4. Pronotum length, 5. Pronotum width, 6. Elytra length, 7. Protibia area, 8. Metatibia length, 9. Prosternum height, and 10. Wing area. Body length was calculated summing pronotum length and elytra length. Wing load was calculated by the ratio of wing area by body size. And volume was calculated by multiplying body size, pronotum width, and prosternum height.

The morphological traits were measured (Figure A3) in five individuals per species and habitat (forest/pasture) in each area, or all captured individuals for species with less than five individuals in each of the sites. That is, we measured traits in up to 10 individuals per species per area, and up to 70 individuals per species per region. To obtain trait measurements, pictures of each individual were taken with a digital camera and using a stereoscope for smaller individuals, and the traits were measured in the software *ImageJ* (Rueden et al., 2017), using a graduated mm paper as a measure reference. Food specificity was measured using Levin's index of niche breadth (Levins, 1968), based on the abundance of individuals of each species in traps with each type of bait of all traps placed in the same region, assuming that the wider the niche, the more generalist is the species.

### 2.4 Functional diversity indices

Trait measurements were used to calculate three functional diversity indices: Functional richness (FRich), functional evenness (FEve), and functional divergence (FDiv) (Mason et al., 2005; Villéger et al., 2008). The indices were calculated for each habitat in each region, using the mean of the five individuals measured from that habitat. FRich measures the functional space of a community and is calculated by the convex hull volume of all the traits of the species present in the community. FEve represents the regularity of abundance of the species in the functional space and is measured by using a minimum spanning tree based on trait similarity between species or individuals. FDiv represents the degree to which the distribution of species in the functional space maximizes the divergence of traits in the community and is calculated by measuring the distance of the species to the centroid of the functional space. All functional indices were calculated using the “FD” function (Laliberté & Legendre, 2010).

### 2.5 Data analysis

#### 2.5.1 Effects of forest conversion on species richness and functional diversity

Taxonomic differences in community structure between habitats (forest and pasture) and between regions (Cerrado and Atlantic Forest) were characterized through principal coordinate analysis (PCoA) sample ordinations. We used generalized mixed models to assess the effects of habitat on species richness and all functional indices, accounting for area differences by including this factor as a random effect, with the following model:

$$\mathrm{FI}\sim \text{Habitat}+\text{Region}+\text{Habitat}\times \text{Region}+\left(1⃓\text{Area}\right)$$

We also calculated the standardized effect size (SES) to remove the effect of species richness in the functional indices. For this, we first created null models by randomizing the community matrices with the function “randomizeMatrix” from the Picante package (Kembel et al., 2010). We set the null model for the “independent swap” algorithm proposed by Gotelli et al. (2011), which maintains species occurrence frequency and sample species richness. The null model was run with 1000 iterations and was replicated 999 times. After randomizing the community, we recalculated FRich, FEve, and FDiv for each permutation. We then calculated SES for all indices using the “ses” function of the “Cati” package (Taudiere & Violle, 2016), setting 0.025 and 0.975 as the confidence interval.

#### 2.5.2 Forest conversion and shift of functional traits in the novel habitats

To assess changes in the functional structure in the novel habitats created by forest conversion, we calculated the community‐weighted mean (CWM; Garnier et al., 2004) of each trait for each assemblage. The CWM combines species trait data with abundance to assess the functional composition of assemblages, weighting the mean trait value of all species in an assemblage by their relative abundances. We used only quantitative traits (Table 1; traits 1–10) to calculate CWM.

#### 2.5.3 Taxonomic and spatial scales influencing dung‐beetle communities

To analyze the effects of habitat on trait variations between and within species (taxonomic scales), we used the decomposition of the variance in nested scales based on restricted maximum likelihood (REML; Messier et al., 2010). This allows assessing which biological scale shows greater variance in the traits.

Furthermore, we used *T*‐statistics (Violle et al., 2012) to understand how internal and external filters (spatial scales) are acting in the assemblages of both types of habitats. *T*‐statistics are ratios of trait variance that measure how this variance is structured across biological and spatial scales. The three ratios calculated in *T*‐statistics are the following:

(1) $$T_{\mathrm{IP}/\mathrm{IC}}=\frac{\sigma_{{\mathrm{IP}}^{2}}}{\sigma_{{\mathrm{IC}}^{2}}},$$

which is the ratio between the within‐population variance of trait values ($\sigma_{{\mathrm{IP}}^{2}})$ and the within‐community variance of trait values $\left(\sigma_{{\mathrm{IC}}^{2}}\right)$, both assessed at the individual level. This ratio measures the strength of internal filtering. It quantifies the overlap of intraspecific trait variation within communities, therefore, measures the niche overlap among coexisting species. The higher its value, the higher the strength of internal filters and the trait overlap among coexisting species.

(2) $$T_{\mathrm{IC}/\mathrm{IR}}=\frac{\sigma_{{\mathrm{IC}}^{2}}}{\sigma_{{\mathrm{IR}}^{2}}},$$

which is the ratio between the within‐communities variance of trait values $\left(\sigma_{{\mathrm{IC}}^{2}}\right)$ and the within regional pool variance of trait values ($\sigma_{{\mathrm{IR}}^{2}})$, both assessed at the individual level regardless of species identity. This ratio measures the strength of external filtering. The higher its value, the lower the strength of external filtering and the higher the trait overlap among communities, at the individual level;

(3) $$T_{\mathrm{PC}/\mathrm{PR}}=\frac{\sigma_{{\mathrm{PC}}^{2}}}{\sigma_{{\mathrm{PR}}^{2}}},$$

which is the ratio between the variance of the population mean trait values within communities ($\sigma_{{\mathrm{PC}}^{2}})$ and the variance of a given species population mean trait values within the regional pool ($\sigma_{{\mathrm{PR}}^{2}})$. This ratio also measures the strength of external filtering, but via population‐level means. The higher the value of $T_{\mathrm{PC}/\mathrm{PR}}$, the lower the strength of external filters at the species level and higher the niche overlap among coexisting species.

To calculate the magnitude of the differences between the observed *T*‐statistic values and those coming from a random assembly of individuals, we estimated the SES (Gotelli & McCabe, 2002) as follows:

$$\mathrm{SES}=\frac{I_{\mathrm{obs}}-I_{\mathrm{sim}}}{\sigma_{\mathrm{sim}}}$$

where $I_{\mathrm{obs}}$ is the *T*‐statistic observed value and $I_{\mathrm{sim}}$ and $\sigma_{\mathrm{sim}}$ are, respectively, the mean value and standard deviation of the null models (*n* = 999 randomizations). The null models were simulated with randomization procedures for each *T*‐statistic. We used the “Cati” package (Taudiere & Violle, 2016) for calculating *T*‐statistics, SES, and generating the null models.

## 3 RESULTS

### 3.1 Effects of forest conversion on species richness and functional diversity

In total, 2681 individuals were captured in our surveys: 2143 in the Cerrado and 538 in the Atlantic Forest, pertaining to 63 species from 18 genera (Table 2, Tables S1 and S2).

**TABLE 2:** Total abundances of the dung beetle species collected in forest and pasture plots of the Cerrado (Goiânia region, Goiás state) and Atlantic Forest (Itajaí Valley, Santa Catarina state).

	Goiânia region (Cerrado)		Itajaí Valley (Atlantic Forest)	
	Forest	Pasture	Forest	Pasture
*Agamopus viridis*		5		
*Ateuchus aff. pruneus*	3			
*Ateuchus vividus*	1			
*Canthidium aff. barbacenicum*		14		
*Canthidium aff. lucidum*	1	1		
*Canthidium aff. trinodosum*			6	
*Canthidium* sp.1	816			
*Canthidium* sp.2	1	1		
*Canthidium* sp.3	1			
*Canthon aff. luctuosos*			1	
*Canthon aff. piluliformis*	1	13		
*Canthon coloratus*			1	
*Canthon conformis*				2
*Canthon curvodilatatus*		2		
*Canthon lituratus*		138		
*Canthon podagricus*			1	4
*Canthon rutilans cyanescens*			229	4
*Canthon* sp.		1		
*Canthonela* sp.	2	5		
*Coprophanaeus bellicosus*			1	
*Coprophanaeus cerberus*			1	
*Coprophanaeus cyanescens*	27			
*Coprophanaeus dardanus*			76	
*Coprophanaeus ensifer*	2			
*Coprophanaeus saphirinus*			10	
*Coprophanaeus spitzi*		1		
*Deltochilum brasilense*			13	
*Deltochilum enceladus*	10			
*Deltochilum furcatum*			28	
*Deltochilum morbilossum*			11	
*Deltochilum multicolor*			26	26
*Deltochilum sextuberculatum*	36			
*Deltochilum* sp.	89			
*Dendropaemon nitidicolis*		1		
*Dichotomius aff. carbonarius*	22	1		
*Dichotomius aff. zicani*	19	3		
*Dichotomius angeloi*	1			
*Dichotomius ascanius*			12	
*Dichotomius bos*	1	28		
*Dichotomius cuprinus*	4			
*Dichotomius mormom*			9	
*Dichotomius nisus*	1	34		
*Dichotomius quadrinodosus*			1	
*Dichotomius sericeus*			20	
*Dichotomius* sp.1	3			
*Dichotomius* sp.2	1			
*Dichotomius transiens*	6			
*Digitontophagus* sp.		27		
*Eurysternus caribaeus*	47			
*Eurysternus nigrovirens*	20			
*Eurysternus paralelus*			19	5
*Eutrichillum hirsutum*	12	1		
*Isocopris inhiatus*		1		
*Ontherus appendiculatus*	1			
*Ontherus asteca*	2			
*Onthophagus aff. hematopus*			20	
*Onthophagus ptox*	326	1		
*Ontophagus buculus*	4	24		
*Phanaeus splendidulus*			4	
*Trichillum adjuntum*	1	5		
*Trichillum externepunctatum*	15	296		
*Trichillum heydeni*		1		
*Uroxys aff. epipleurysternusalis*	57			

**FIGURE 2:**
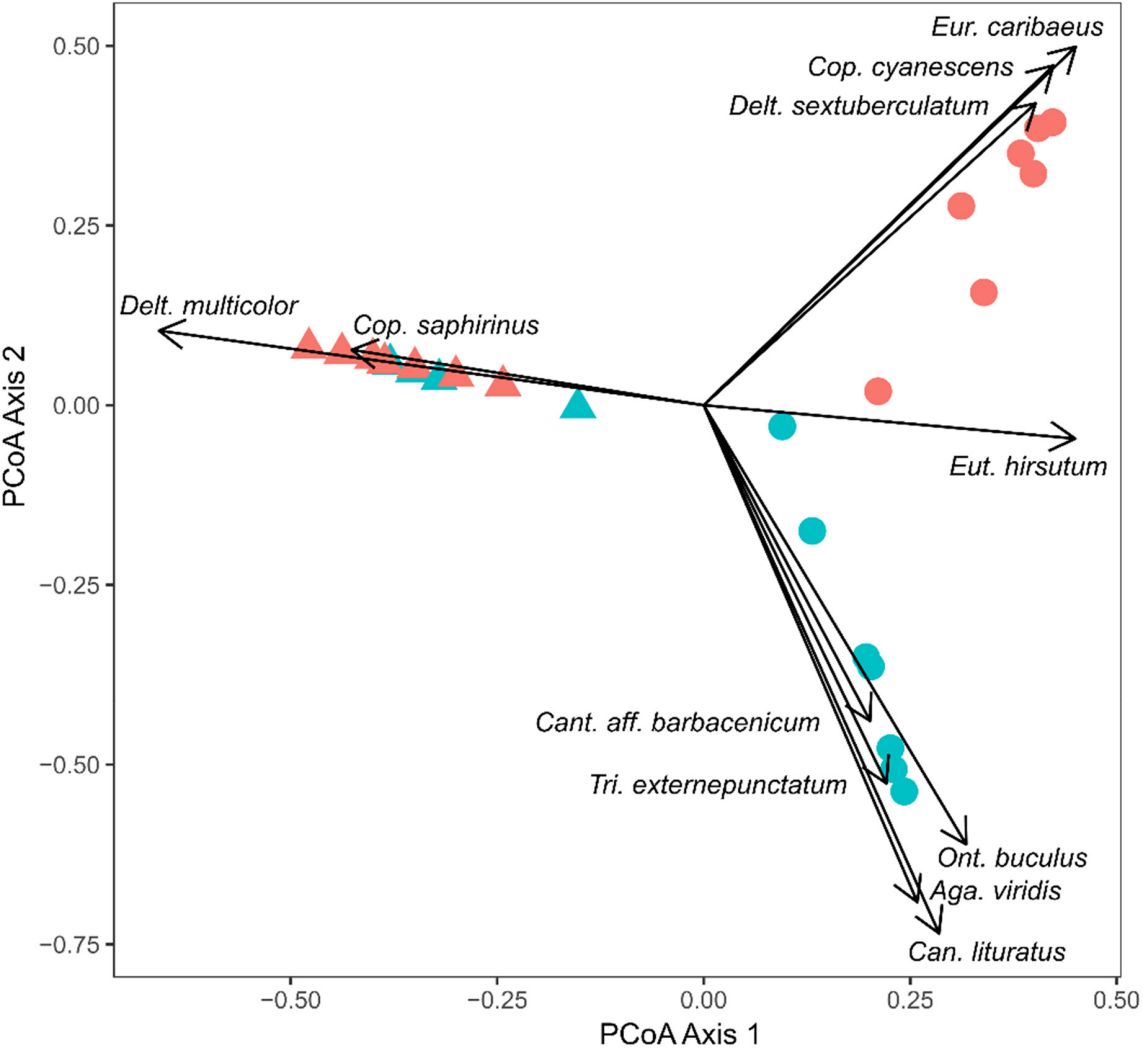
Pcoa Axis for the dung beetles surveyed in forest and pasture habitats of the Goiânia region and the Itajaí Valley (placed at the Cerrado and Atlantic Forest biomes, respectively). Circles represent Goiânia region, and triangles represent Itajaí Valley. In red Forest and in green Pasture.

**FIGURE 3:**
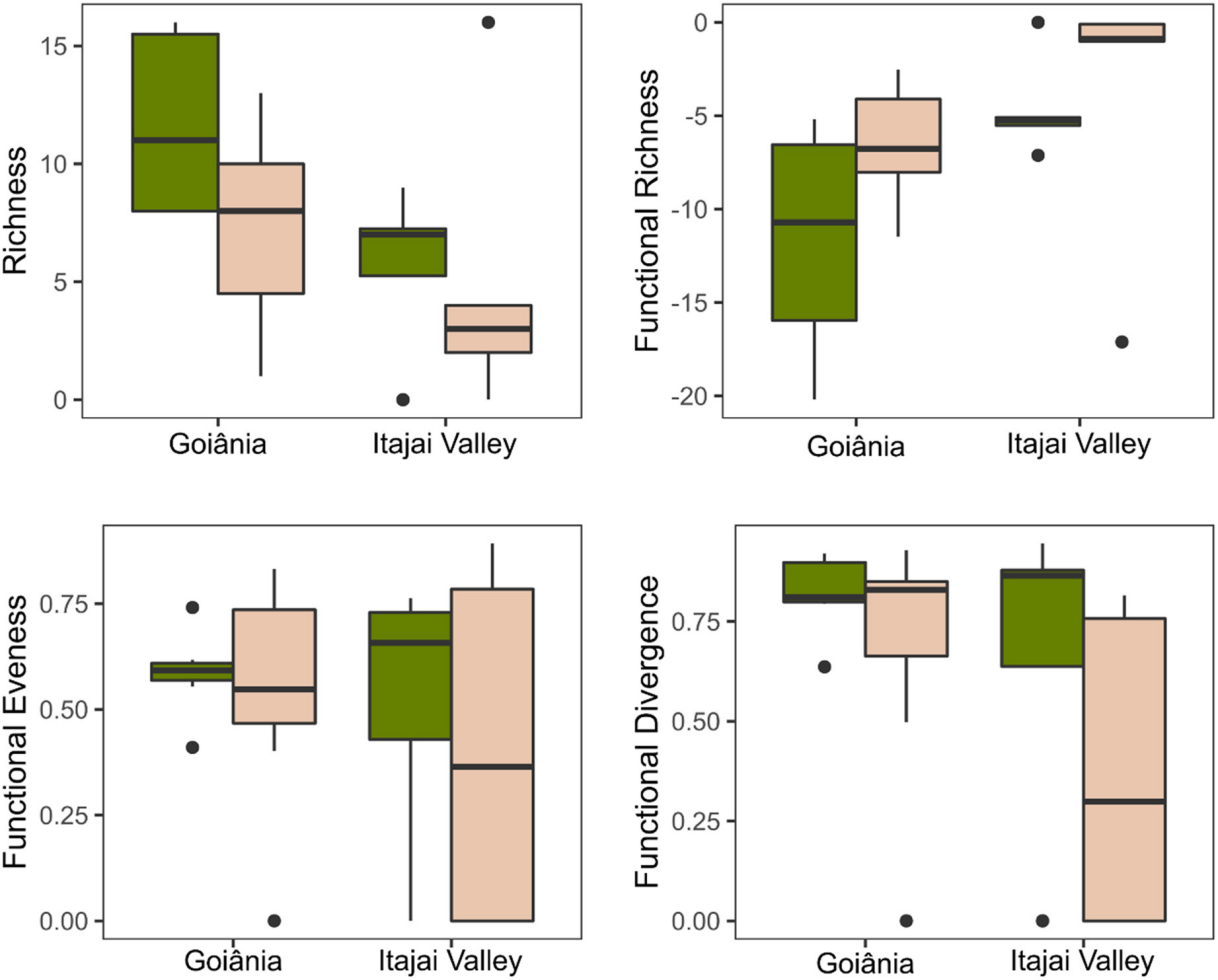
Dung beetle species richness and functional diversity in forest and pasture habitats of the Goiânia region and the Itajaí Valley (placed at the Cerrado and Atlantic Forest biomes, respectively). Box plots show the average and interquartile range of site values; dots identify extreme values. Green boxes represent forests and beige pasture communities.

In the Atlantic Forest, the most common species were *Canthon rutilans cyanescens* Harold, 1868 (43% of all captured individuals), *Coprophanaeus dardanus* (MacLeay, 1819) (15%), and *Deltochilum multicolor* Balthasar, 1939 (9%), whereas *Canthidium* sp.1 (61%), *Onthophagus ptox* Erichson, 1842 (25%), and *Trichillum externepuctatum* Preudhomme de Borre, 1880 (23%) were so at the Cerrado. We found a regional effect and a marginal habitat effect on dung beetle richness (Table 3). The Goiânia region was richer, with 42 species compared to the 21 species found at the Itajaí Valley. Forest habitats hosted more species than pastures in both regions (32 vs. 23 species in Cerrado, and 20 vs. 6 species at the Atlantic Forest). In the Atlantic Forest, only one species was exclusive from pastures and 15 were so from the forest, whereas the Cerrado region have 11 species exclusive to pastures and 20 exclusives to the forest. Results from a Principal Coordinates Analysis (PCoA) from species abundance data evidence the differentiation in the species pools of both regions (Figure 2). But also, that species composition differs clearly between forests and pastures in the Cerrado, while these habitat differences are smaller for the Atlantic Forest, since sites from both types of habitats largely overlap in these PcoA Axes (Figure 2). None of the functional indices were related to forest conversion, even when removing the effects of richness and abundance by calculating the SES (Figure 3 and Table 3). Nonetheless, when traits are analyzed individually, we found regional effects in all traits except for the eye dorsal area and volume (Table 4).

**TABLE 3:** Results of the linear mixed models for the effects of habitat and region (and their interaction) on species richness and functional diversity indices, and their standardized effect sizes (SES).

	Model	Value	Std. error	df	*t*‐Value	*p*‐Value
S	Habitat	**−4.43**	**2.30**	**13**	**−1.92**	**.08**
	Region	**−6.09**	**2.23**	**11**	**−2.73**	**.02**
	Habitat × Region	3.47	3.28	13	1.06	.31
FRich	Habitat	0.00	0.08	13	0.01	1.00
	Region	0.17	0.14	11	1.19	.26
	Habitat × Region	0.15	0.12	13	1.26	.23
FEve	Habitat	−0.04	0.16	13	−0.27	.79
	Region	−0.07	0.16	11	−0.44	.67
	Habitat × Region	−0.07	0.23	13	−0.29	.78
FDiv	Habitat	−0.14	0.18	13	−0.77	.45
	Region	−0.16	0.17	11	−0.92	.38
	Habitat × Region	−0.15	0.26	13	−0.59	.57
FRichSES	Habitat	−0.01	0.37	7	−0.03	.97
	Region	−0.49	0.55	11	−0.88	.40
	Habitat × Region	0.28	0.63	7	0.44	.67
FEveSES	Habitat	0.56	0.52	7	1.08	.32
	Region	−0.61	0.52	11	−1.17	.27
	Habitat × Region	0.55	0.84	7	0.66	.53
FDivSES	Habitat	−0.56	0.47	7	−1.19	.27
	Region	−0.65	1.21	11	−0.54	.60
	Habitat × Region	0.31	0.81	7	0.39	.71

*Note*: S stands for species richness, FRich for functional richness, FEve for functional evenness, and FDiv for functional divergence. Significant and nearly significant models are highlighted in bold.

**TABLE 4:** Results of the linear mixed models of the community‐weighted mean of individual traits.

	Model	Value	Std. error	df	*t*‐Value	*p*‐Value
Wing load	Habitat	**1.59**	**0.77**	**11**	**2.07**	**.06**
	Region	**2.29**	**1.05**	**10**	**2.18**	**.05**
	Habitat × Region	**−2.37**	**1.17**	**11**	**−2.02**	**.07**
Eye dorsal area	Habitat	0.22	0.16	11	1.38	.20
	Region	0.26	0.19	10	1.38	.20
	Habitat × Region	−0.24	0.24	11	−0.99	.34
Prosternum height	Habitat	0.86	0.57	11	1.49	.16
	Region	**2.27**	**0.98**	**10**	**2.32**	**.04**
	Habitat × Region	−1.25	0.88	11	−1.41	.18
Protibia area	Habitat	1.70	1.03	11	1.64	.13
	Region	**2.83**	**1.30**	**10**	**2.18**	**.05**
	Habitat × Region	−1.93	1.57	11	−1.23	.25
Pronotum width	Habitat	1.31	0.90	11	1.45	.17
	Region	**4.37**	**1.37**	**10**	**3.18**	**.01**
	Habitat × Region	−1.05	1.38	11	−0.77	.46
Head length	Habitat	0.59	0.40	11	1.48	.17
	Region	**1.19**	**0.53**	**10**	**2.24**	**.05**
	Habitat × Region	−0.70	0.60	11	−1.15	.27
Head width	Habitat	0.82	0.54	11	1.51	.16
	Region	**2.45**	**0.83**	**10**	**2.96**	**.01**
	Habitat × Region	−0.72	0.83	11	−0.88	.40
Body size	Habitat	0.83	1.00	11	0.83	.42
	Region	**5.87**	**1.81**	**10**	**3.24**	**.01**
	Habitat × Region	0.67	1.54	11	0.44	.67
Volume	Habitat	292.91	217.54	11	1.35	.21
	Region	**577.06**	**317.36**	**10**	**1.82**	**.10**
	Habitat × Region	−293.02	332.67	11	−0.88	.40
Metatibia length	Habitat	0.12	0.24	11	0.50	.63
	Region	**2.44**	**0.53**	**10**	**4.62**	**.00**
	Habitat × Region	0.53	0.37	11	1.44	.18
Standardized Levins	Habitat	−0.05	0.04	11	−1.32	.22
	Region	**−0.12**	**0.05**	**10**	**−2.31**	**.04**
	Habitat × Region	**0.13**	**0.06**	**11**	**2.18**	**.05**

*Note*: Significant or nearly significant results are highlighted in bold.

### 3.2 Forest conversion and shift of functional traits in the novel habitats

**FIGURE 4:**
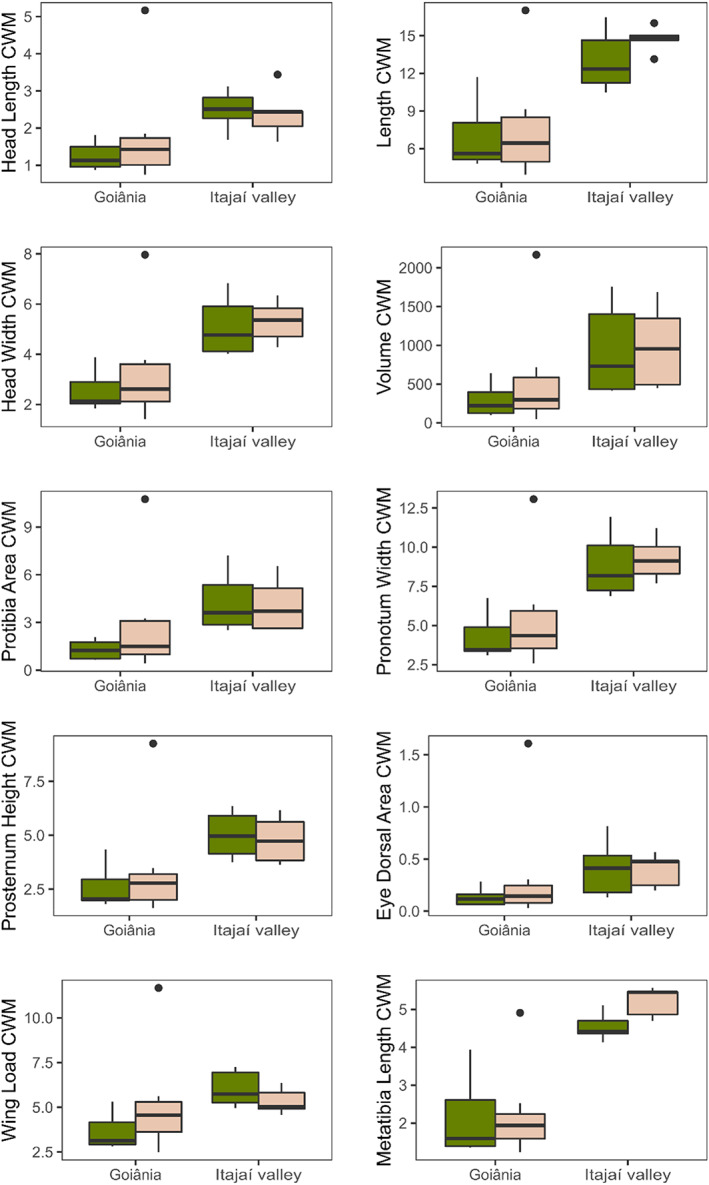
Dung beetle CWM in forest and pasture habitats of the Goiânia region and the Itajaí Valley (placed at the Cerrado and Atlantic Forest biomes, respectively). Box plots show the average and interquartile range of site values; dots identify extreme values. Green boxes represent forests and beige pasture communities.

The analyses on individual traits obtained by CWM analysis showed that the values of most of them differ between regions, with the Atlantic Forest (Table S3) presenting larger values and greater variance in the community‐weighted mean for all continuous traits (Table 4; Figure 4). In contrast, habitat type only showed nearly significant effects on wing load.

### 3.3 Taxonomic and spatial scales influencing dung‐beetle communities

**FIGURE 5:**
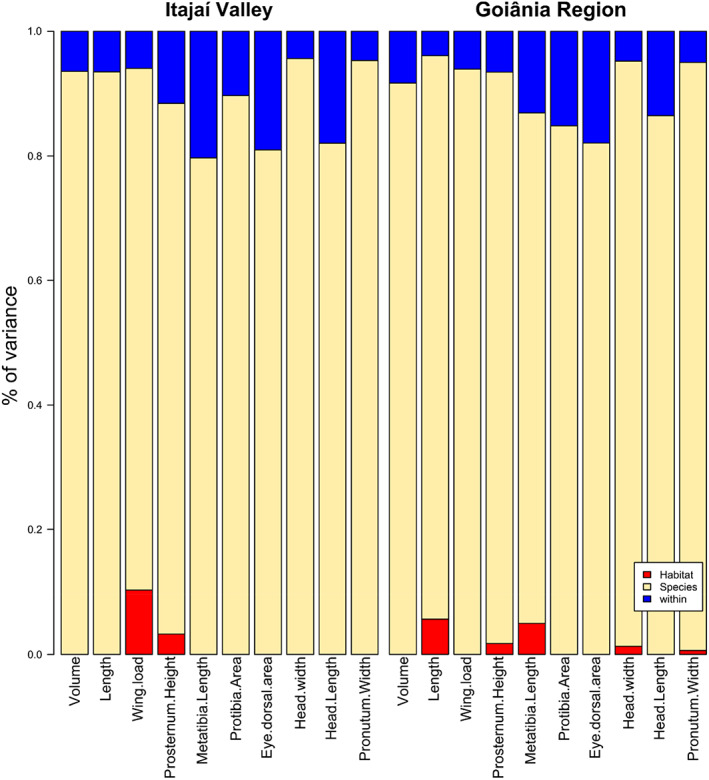
Nested partition of dung beetle trait variance surveyed in forest patches and pastures in Atlantic Forest and Cerrado.

**FIGURE 6:**
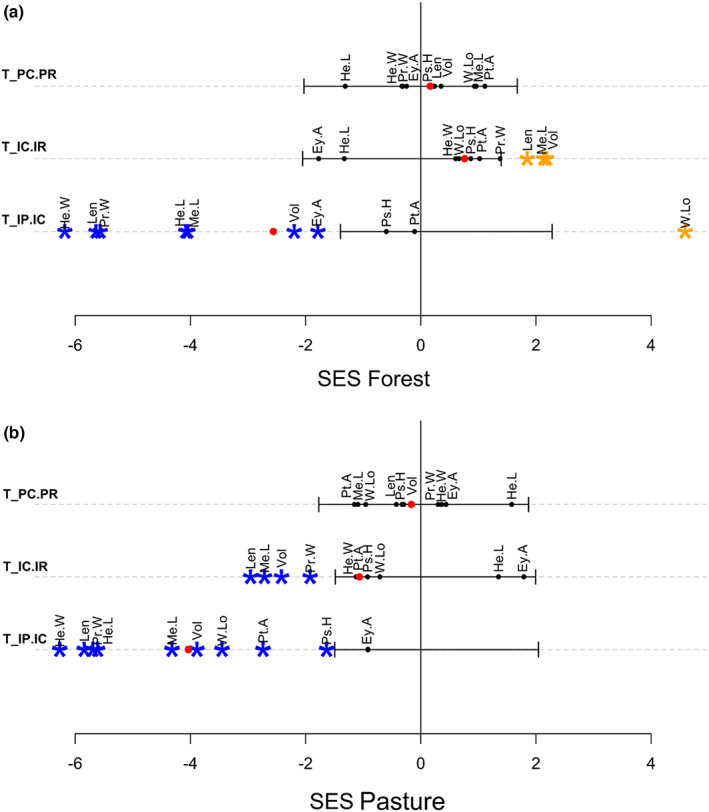
Standardized effect size of Trait Statistics obtained for 10 dung beetle traits in Cerrado forest patches (a) and pastures (b). The solid lines indicate the confidence interval of the null model for all traits to each *T*‐statistic. Red dots indicate the average SES of all traits for each *T*‐statistic. Blue and orange asterisks, respectively, represent values significantly lower and higher than expected compared to the null models (*p* < .05). Ey.A, eye dorsal area; He.L, head length; He.W, head width; Len, length; Me.L, metatibia length; Pr.W, pronotum width; Ps.H, prosternum height; Pt.A, protibia area; T_IC.IR, external filtering of individuals; T_IP.IC, internal filtering of individuals; T_PC.PR, external filtering of species; Vol, volume; W.Lo, wing load.

**FIGURE 7:**
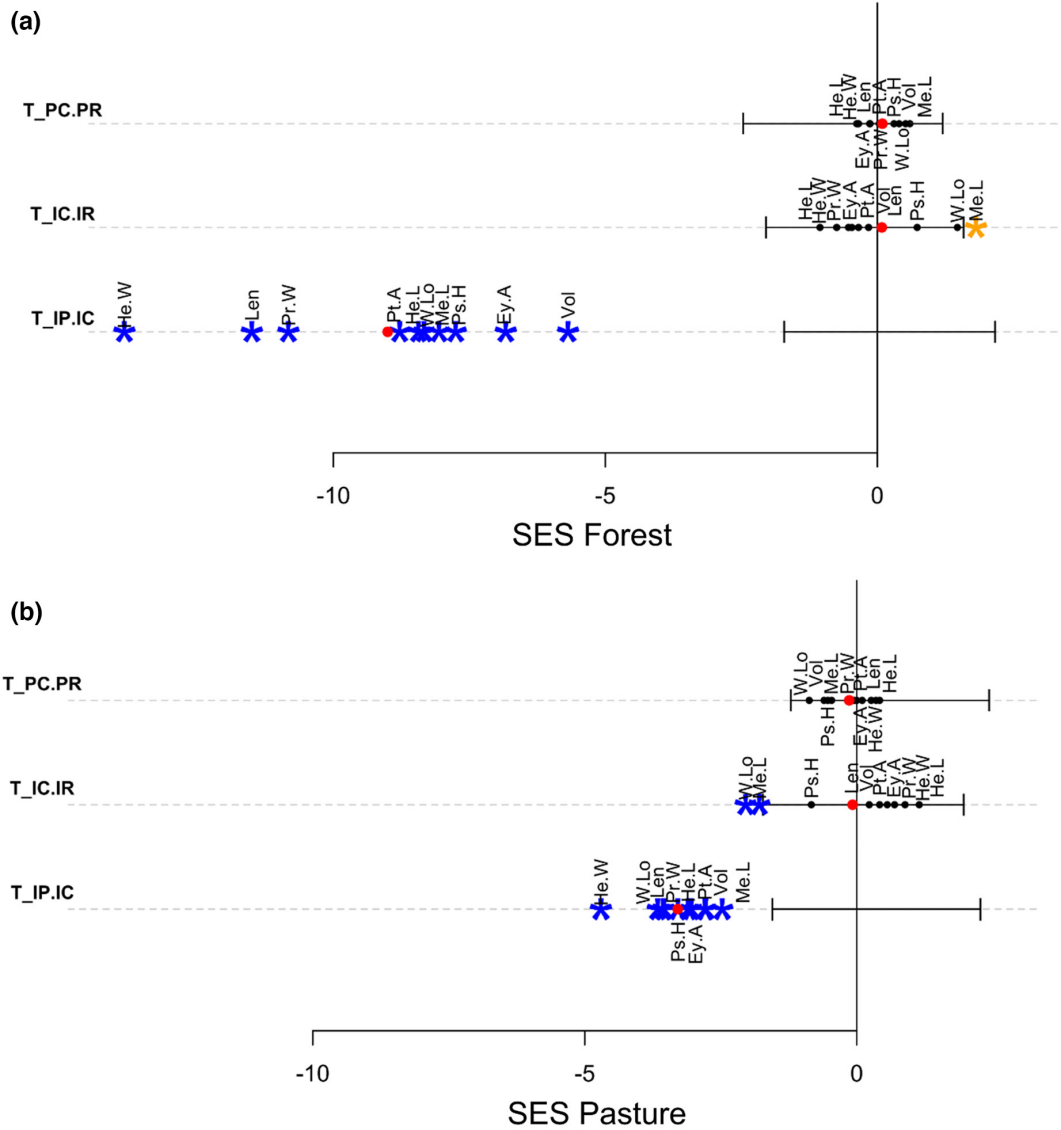
Standardized effect size of Trait Statistics obtained for each dung beetle trait in Atlantic Forest forest patches (a) and pastures (b). The black solid lines indicate the confidence interval of the null model for all traits to each *T*‐statistic. Red dots indicate the average standardized effect size of all traits for each *T*‐statistic. Blue and orange asterisks respectively represent values significantly lower and higher than expected compared to the null models (*p* < .05). Ey.A, eye dorsal area; He.L, head length; He.W, head width; Len, length; Me.L, metatibia length; Pr.W, pronotum width; Ps.H, prosternum height; Pt.A, protibia area; T_IC.IR, external filtering of individuals; T_IP.IC, internal filtering of individuals; T_PC.PR, external filtering of species; Vol, volume; W.Lo, wing load.

Habitat contributed little to the nested variance of traits, and the differences between species were the principal factor that promoted variance in both regions (Figure 5). Trait Statistics partly corroborated the results obtained by CWM: all traits in the Atlantic Forest and almost all traits in the Cerrado exhibited lower trait variations than null models in both habitats (Figures 6 and 7), emphasizing the importance of internal filters shaping the dung beetle community in both regions and habitats. The effects of external filtering, expressed by $T_{\mathrm{IC}/\mathrm{IR}}$, were more variable. Most of the traits had values that not differed from null models; meanwhile, traits that presented values of $T_{\mathrm{IC}/\mathrm{IR}}$ lower than expected in pasture had values greater than expected in forest. In contrast, values of $T_{\mathrm{PC}/\mathrm{PR}}$ did not differ from expected by chance for all traits for both regions (Figures 6 and 7).

## 4 DISCUSSION

Our results show that the conversion of forest to pasture affected mainly species composition both in the Atlantic Forest and the Cerrado. Strikingly, although the overall functional structure of the communities was apparently not affected by habitat changes, the decomposition of these effects by traits points significant changes between regions and habitats. This is especially true for intraspecific variation, as the variance of traits between habitats and regions comes from species differences, fostered by internal (i.e., within species) filtering in almost all traits, with a small contribution of external filtering processes that affected only a few traits.

Although in both regions, the novel pastures are poorer in species and host individuals with different trait values than the native forest habitats, the conversion of forest habitats into pastures affected only dung beetle species richness. We expected that the more recent conversion at the Cerrado would have resulted in poorer pasture communities than in the Atlantic Forest, where native dung beetles have had more time for colonizing the novel habitat. However, the pattern is the opposite: Atlantic Forest pasture assemblages are poor and dominated by a handful of alien species, whereas a large number of native species have been able to colonize the new open areas at the Cerrado. In any case, the differences between biomes due to differences in their species pools are apparent beyond the raw effects of habitat change. In biomes without open habitat species, pastures and cleared secondary forests are colonized by generalist forest species or by exotic species from other regional pools, such as in the Atlantic Forest example shown here, or the pastures in Amazonian regions, that are colonized mostly by limited numbers of Cerrado and Chaco species (Silva et al., 2014), resulting in the diminished ecosystem functioning in perturbed forests of this biome (Noriega, March‐Salas, et al., 2021). In the Cerrado, the novel habitat is colonized by species that were already adapted to utilize the (semi)open habitats of the Brazilian savannah. In contrast, in the Atlantic Forest of the Itajaí Valley, where there were no natural open habitats, the only exclusive pasture species were rare and the pasture is currently used only by a handful of generalist native Atlantic Forest species and invaders. These differences in the effect of, arguably, the same type of habitat filtering are the consequence of the ecological and evolutionary differences throughout the historical formation of the pool of the two regional communities (sensu Ricklefs, 2015).

In the Argentinian forests, the regional context reflected in different degrees of impact of forest conversion on dung beetle communities, where humid forests presented a higher impact on dung beetle functional diversity than dry forests (Guerra Alonso et al., 2022). This contrasts with our results, where these regional differences did not reflect directly on the functional diversity of dung beetle communities. When the differences due to richness are removed (i.e., by using the Standardized Effect Size; Gotelli & McCabe, 2002), functional shifts between both types of habitats are relatively small, in apparent contrast with the large functional losses found in novel habitats of other regions of the Neotropics (e.g., Argentinian Atlantic Forest, Gómez‐Cifuentes et al., 2017; Argentinian pastures, Giménez Gómez et al., 2022; Mexican rainforest, Barragán et al., 2011; El Salvador tropical dry forest, Horgan, 2008; Brazilian Pantanal, Pessôa et al., 2017; Colombian Andean, Amazonian and Caribbean forests, Noriega, March‐Salas, et al., 2021). This generalized loss of functionality has been attributed to the smaller diversity of resources (Lumaret et al., 1992) and/or changes in microclimatic conditions (Gómez et al., 2018). The contrastingly smaller losses of trait variations found in our analysis compared with these studies may be due to our use of more continuous traits and individual trait measurements, which may have diluted functional effects. But also, the fact that, once the loss of species is accounted for, pasture species presented so extreme values of traits that functionality was maintained. In fact, open habitats may even increase the diversity of physiological response traits, accounting for the more extreme (micro)climatic conditions of pastures compared to forests (Giménez Gómez et al., 2022). Besides that, the reduced loss of functional diversity in some biomes may be due to a high functional redundancy in their pool of dung beetle species, which allows maintaining the functionality in each type of habitat despite the regional changes in species composition. This difference between species is observed in the effects on trait community weighted means. All traits presented only a regional effect (evidencing the differences in the functional solutions present in the pool of each biome), while only wing load presented a marginal difference between pasture and forest. All traits presented a higher CWM in the Atlantic Forest, due to greater habitat heterogeneity presented in this habitat. The lack of difference in the CWM of the traits contrasts Guerra Alonso et al. (2022) findings that traits related to size and food relocation presented clear differences between forest and pasture, mainly due to forest energy restrictions to telecoprids and the consequence dominance of this habitat by paracoprids.

The partition of trait variance shows that interspecific variation had a greater contribution for the total variance of traits in both regions, corroborating the results found both in dung beetles (Griffiths et al., 2016) and other groups (De Bello et al., 2009; Messier et al., 2010). However, even though traits vary more between than within species, our results indicate significant intraspecific variation between habitats. In fact, our results show no effects of filters when ignoring intraspecific variation (the metric $T_{\mathrm{PC}/\mathrm{PR}}$), while showing signal of external filtering for some traits when considering intraspecific trait variation (the metric $T_{\mathrm{IC}/\mathrm{IR}}$). This result emphasizes the importance of considering intraspecific variation in community studies (MacArthur & Levins, 1967; Violle et al., 2012).

Eye Dorsal Area in both regions, and Metatibia and Head Length in the Itajaí Valley, presented a slightly higher intraspecific contribution than other traits, although still much smaller than interspecific variation. Eye dorsal area can be related to both flight ability, a period of daily activity, and the adaptation to different light conditions (Byrne & Dacke, 2011). While in both regions this trait presented low CWM, it presented a greater variance in the forest. This may be due to the presence of species adapted to closed areas suffering filtering of individuals with certain trait characteristics, thus increasing the phenotypic diversity of this trait within species. Indeed, the pasture presents a greater influence of light than forests, which can present greater differences in the eye structure of diurnal and nocturnal species.

Metatibia length presented differences mostly between regions. In the Atlantic Forest, pasture communities presented shorter metatibias, because the generalist species that dominate the open habitat are smaller, even despite the dominance of roller species. In contrast, Cerrado communities were characterized by more dwellers and smaller species than those of the Atlantic Forest, presenting no differences in CWM between habitats, though greater variance in the forest probably because of the highest number of rollers and bigger species than in the pasture. In this biome, we also found intraspecific variance in the prosternum height and protibia area, two related to excavation (deCastro‐Arrazola et al., 2023; Halffter & Matthews, 1966). Indeed, soil texture and compaction affect the assembly of dung beetle communities (Davis, 1996). Therefore, the uneven compaction of the soil in the pasture may be selecting a larger interspecific variance in these traits, through the selection of individuals adapted to exploit soils both well‐developed soils and those that have been compacted by cattle.

The greater promoter of individual variance in our data is internal filtering, which is consistent with other studies that use *T*‐statistics (Luo et al., 2016; Mungee & Athreya, 2021; Xavier Jordani et al., 2019; Zorger et al., 2019). The metric $T_{\mathrm{IP}/\mathrm{IC}}$ was lower than expected by chance for almost all traits, suggesting little niche overlap, which can be promoted by local processes such as competition. Indeed, those strong internal filtering effects were expected, since dung beetles present highly competitive communities due to the use of an ephemeral resource (sensu Atkinson & Shorrocks, 1981; Elton, 1966), which may be even stronger in the pasture considering that microclimatic conditions of the dung pat diminish the opportunity window of resource availability.

Several traits show a signal of external filtering, presenting opposing patterns in the two habitats. While the external filtering processes of the forest promoted overdispersion, increase in niche overlap, in the pasture they promoted clustering, niche packeting. In the pasture, the fluctuation of heat and humidity may impose an important filter for selecting species and individuals with particular trait values. While in the forest the greater environmental stability promotes heterogeneity in the traits and the persistence of more strategies for resource utilization. At the Cerrado, forest habitats increase the individual variation of Body Length and Volume, and metatibia length, while in the pasture, the individual variation in those traits and pronotum width decreases. In the case of metatibia length, a trait related to the ability to roll dung balls (Halffter & Matthews, 1966; Hanski & Cambefort, 1991), this effect may be due to the lower presence of rollers in the forest (Krell et al., 2003). The dominance of tunnelers and dwellers in the forest may increase the individual variation in this trait, in contrast to the dominance of rollers in the pasture. Length, Volume, and Pronotum width represent different aspects of body size. Finally, individual variation in size may determine the amount of resources utilized for development (Emlen et al., 2005). Therefore, the greater variation in the forest may be a reflection of the uneven availability of resources in contrast with the greater presence of cow dung in the pasture. This fact, in addition to a competition promoted by ephemeral resources, can lead to filtering processes in the pasture that promoted niche differentiation, while in the forest we found processes increasing niche packing and overlap.

## 5 CONCLUDING REMARKS

The contrasting results of our work with other studies regarding richness and functional diversity emphasizes the complementarity of both diversity components, since we found similar results of higher impacts of forest conversion in humid forests than in dry forests, but on another aspect of dung beetle diversity. Besides that, our work evidence that it is necessary to consider intraspecific variation to acount for all assembly processes and filtering mechanisms operating over ecological communities subject to rapid habitat shifts, as we did not find effects of forest conversion in neither the indices nor the external filtering of Trait Statistics that do not consider individual variance. This may be accentuated by the fact that competition for ephemeral resources may be stronger both at the species and the individual level in novel habitats. Including intraspecific variation increase our understanding of those processes shaping the communities under rapid global change.

To summarize, forest conversion into pasture impoverishes the diversity of dung beetle communities of the Cerrado and the Atlantic forest. However, the characteristics of the particular species available in the pool of each biome may diminish this effect, since the ability to colonize the novel habitat depends on the presence of species either previously adapted to this environment, or showing larger phenotypic plasticity. In regions where the pool of species is poor in species adapted to open areas, time since the land clearance is not important for dung beetle community regeneration. Importantly, trait filtering occurs independently of the presence of species previously adapted to the new environment. Internal filtering presents a strong effect in all regions and habitats, even though we also found external filtering in some traits. Rather, differences between regions and habitats on the external filtering of communities could be accessed only when individual variance was considered, showing the importance of individual variance in the functional responses of dung beetle communities to forest conversion.

## AUTHOR CONTRIBUTIONS

**Marcelo Bruno Pessôa:** Conceptualization (equal); data curation (equal); formal analysis (equal); investigation (equal); methodology (equal); writing – original draft (lead); writing – review & editing (equal). **Tatiana Souza do Amaral:** Formal analysis (equal); investigation (equal); methodology (equal); writing – original draft (equal); writing – review and editing (equal). **Paulo De Marco Júnior:** Conceptualization (equal); methodology (equal); supervision (equal); writing – review and editing (equal). **Joaquín Hortal:** Conceptualization (equal); formal analysis (equal); funding acquisition (lead); investigation (equal); methodology (equal); supervision (equal); visualization (equal); writing – original draft (equal); writing – review & editing (equal).

## CONFLICT OF INTEREST STATEMENT

The authors declare no conflict of interest.

## Supporting information


Tables S1–S8

## Data Availability

The data of the present work can be found as supporting information in the online version of this paper.
